# Electron and proton FLASH beam dosimetry using unified alanine, EBT‐XD, and HD‐V2 Gafchromic film dosimeters

**DOI:** 10.1002/mp.70022

**Published:** 2025-09-25

**Authors:** Seongmoon Jung, In Jung Kim, Chul‐Young Yi, Yun Ho Kim, Young Min Seong, Rukundo Solomon, Sang Hyoun Choi, Young‐jae Jang, Se Byeong Lee, Chae‐Eon Kim, Sang‐il Pak, Jong In Park

**Affiliations:** ^1^ Ionizing Radiation Metrology Group Korea Research Institute of Standards and Science Daejeon Republic of Korea; ^2^ Precision Measurement Engineering University of Science and Technology Daejeon Republic of Korea; ^3^ Radiation Therapy Technology and Standards, Korea Institute of Radiological & Medical Sciences Seoul Republic of Korea; ^4^ Proton Therapy Center, National Cancer Center Goyang Republic of Korea; ^5^ Department of Nuclear Engineering Hanyang University Seoul Republic of Korea; ^6^ Department of Radiation Oncology Pusan National University Yangsan Hospital Yangsan Republic of Korea

**Keywords:** alanine dosimeter, EBT‐XD film, electron FLASH, HD‐V2 film, proton FLASH, radiotherapy

## Abstract

**Background:**

Ultra‐high dose rate (UHDR) radiotherapy, or FLASH RT, has shown potential to spare normal tissues while maintaining tumor control. However, accurate dosimetry at UHDR remains challenging, as conventional ionization chambers suffer from recombination effects. Although radiochromic films and alanine dosimeters have both been investigated independently for FLASH dosimetry, their separate use hinders robust validation and direct comparison of their measurements.

**Purpose:**

This study aims to develop and evaluate a unified dosimeter containing both alanine and radiochromic film for electron and proton FLASH beam dosimetry. The design allows for simultaneous, co‐located irradiation of both dosimeter types, enabling a direct comparison between them. This configuration eliminates confounding factors such as positional offsets, alignment errors, and beam fluctuations, thereby facilitating the validation of measurements and enhancing confidence in FLASH dosimetry.

**Methods:**

The unified alanine and EBT‐XD/HD‐V2 film dosimeter was designed with the same outer dimensions as the Advanced Markus chamber (PTW‐Freiburg), allowing compatibility with commercial QA phantoms. Alanine and film dosimeters were calibrated under conventional electron and proton beams, traceable to absorbed dose to water from Co‐60 gamma rays. The unified dosimeter was used to measure dose from a 9 MeV electron FLASH beam (Varian Clinac iX) and a 230 MeV proton FLASH beam (IBA machine), with alanine and film irradiated simultaneously at the same location.

**Results:**

The alanine dosimeter measured the dose per pulse, instantaneous dose rate, and mean dose rate at a source‐to‐surface distance of 100 cm for the electron FLASH beam as 0.99 ± 0.02 Gy/pulse, 2.48 × 10^5^ Gy/s, and 357 Gy/s, respectively. The EBT‐XD film showed good agreement (within a 2.0% relative difference) in the 10–30‐Gy range, whereas the HD‐V2 indicated a larger difference (up to 5.9%) compared to the alanine dosimeter. The mean dose rate for the proton FLASH beam, measured by the alanine dosimeter, was 115.4± 1.1 Gy/s. The EBT‐XD showed a 4.3% relative difference with the alanine dosimeter in the 10–30‐Gy range.

**Conclusions:**

The unified alanine and film dosimeters enabled simultaneous irradiation of the alanine and the films, with combined relative standard uncertainties of 2.4% (*k* = 1) for the alanine dosimeter and 3.5% (*k* = 1) for the EBT‐XD films at the electron FLASH beam. For the proton FLASH beam, these uncertainties were 3.2% (*k* = 1) for both the alanine dosimeter and the EBT‐XD films. Until dosimetry guidelines for the FLASH RT community are established by a working group such as AAPM TG‐359, the dosimetry protocol proposed in this study can serve as a promising approach for FLASH RT facilities worldwide.

## 1 INTRODUCTION

Numerous studies have reported the potential use of ultra‐high dose rate (UHDR) radiotherapy (RT), also known as FLASH RT. FLASH RT delivers the dose at UHDR (averaged dose rate ≥ 40 Gy/s).^1^, ^2^, ^3^, ^4^, ^5^, ^6^, ^7^, ^8^, ^9^, ^10^, ^11^ It has been shown to reduce damage to healthy tissues while maintaining its therapeutic effect on cancer cells in animal studies.^1^, ^12^ To evaluate the efficacy of FLASH RT compared with conventional RT (denoted as CONV RT), accurate measurement of the absorbed dose to water at UHDR is essential. Ionization chambers have been employed for FLASH RT dosimetry, as they serve as the reference dosimeter for CONV RT. However, accurate dosimetry with ionization chambers has been hampered by significant recombination effects.^13^, ^14^ Consequently, alternative dosimeters, such as diamond detectors, thermoluminescence dosimeters (TLDs), optically stimulated luminescence dosimeters (OSLDs), radiochromic films, and alanine dosimeters, have been widely introduced for FLASH RT dosimetry.^12^, ^15^, ^16^, ^17^, ^18^, ^19^, ^20^, ^21^, ^22^ These dosimeters are recognized for their independence from dose rate, making them suitable for use at UHDR.^23^, ^24^

Among these dosimeters, radiochromic films have been the most commonly used for FLASH RT dosimetry. Various models of radiochromic films were produced with differing sensitivities to dose and dose dynamic range. For FLASH RT applications, the dosimetric characteristics of External Beam Therapy (EBT3), EBT‐XD, and HD‐V2 films (Ashland Specialty Ingredients G.P., Bridgewater, NJ, USA) have been studied.^12^, ^15^, ^16^, ^17^, ^18^, ^19^, ^20^, ^21^, ^22^ Some studies have compared the dose measured by these films with that measured by ionization chambers,^18^, ^21^ while others have used a third dosimeter (e.g., TLD, OSLD, or alanine) to validate the dose measured by radiochromic films or ionization chambers.^12^, ^15^, ^16^, ^17^, ^19^, ^22^ Although radiochromic films offer advantages such as dose rate independence and ease of handling, film dosimetry is associated with relatively high uncertainty. Therefore, the use of alanine dosimeters has been proposed by several groups.^12^, ^15^, ^22^

The reported relative standard uncertainty of the EBT‐XD film was approximately 3.1% (*k* = 1) for doses ranging from 5 to 40 Gy using 6 MV CONV X‐rays.^25^ More recently, Szpala et al. utilized EBT‐XD film for UHDR electron beams up to 2×10^4^ Gy/s, although limited information on dose uncertainty was provided.^17^ The EBT‐XD has also been employed for proton FLASH beams.^18^ Similarly, the HD‐V2 film has been used for dosimetry of UHDR electron or proton beam.^19^, ^20^, ^21^ Korysko et al. conducted dosimetry using the HD‐V2 film alongside radio‐photoluminescence dosimeters,^19^ and Dal Bello et al. commissioned a UHDR machine using EBT3 in the 0−20‐Gy and HD‐V2 in the 0−100‐Gy range.^21^ However, none of these studies thoroughly evaluated the uncertainty in the measured dose or provided a clear link to a reference dose that ensures traceability to the National Metrology Institute (NMI).

Conversely, the alanine dosimeter is known to have the least standard uncertainty and minimal energy dependency compared to other dosimeters, such as TLDs, OSLDs, and radiochromic films, while also being independent of dose rate. Several studies have already used alanine dosimeters for FLASH RT dosimetry, including preclinical studies and the first human treatment case.^2^, ^26^, ^27^, ^28^ The overall uncertainty in alanine dose measurement was found to be 5.1% (*k* = 2).^26^, ^28^ In 2022, a group from CHUV and Stanford developed a promising acrylic cuboid phantom (25 × 25 × 32 mm^3^), enabling the simultaneous irradiation and robust validation of six EBT3 films, three TLDs, and two alanine pellets, all housed in a 5‐mm‐diameter, 10.4‐mm‐length cylindrical cavity.^28^ This cuboid phantom, containing three types of dosimeters, was used to validate dosimetry across multiple electron FLASH RT facilities.^28^ Furthermore, the alanine dosimeter has been used as a transfer standard for intercomparison between the PTB and METAS primary standards in FLASH electron beams. However, establishing an accurate alanine dosimetry system requires specialized expertise, equipment, and significant time, limiting its accessibility to most hospitals and research institutions.^13^

Previous studies that incorporate two or more types of passive dosimeters (e.g., radiochromic film, TLD, OSLD, and alanine dosimeters) typically validate measurements through separate irradiations.^16^, ^17^, ^19^, ^26^ However, this approach introduces uncertainties from beam fluctuations, positional non‐uniformity, and setup differences when comparing measurements. To overcome these challenges, we propose a unified dosimeter capsule that integrates alanine pellets and films in the same geometry, allowing co‐located and simultaneous irradiation under identical FLASH electron or proton beam conditions. This approach minimizes uncertainties associated with separate irradiations and enables direct and robust dose comparison between film and alanine.

Although some studies have validated the measurements from alanine and radiochromic films during in vivo experiments with simultaneous irradiation,^12^, ^15^, ^22^ these studies primarily focus on measuring doses in vivo, rather than on making a robust comparison between two dosimeter types. Additionally, surface dosimetry increases uncertainty due to differences in measurement environments compared to reference dosimetry setups. Factors such as high dose gradients, lack of charged particle equilibrium, and non‐uniform surfaces complicate dose comparisons and hinder the accuracy of measurements.

In this study, we aim to perform dosimetry for electron and proton FLASH beams employing the unified alanine and radiochromic film dosimeter. The geometry of the unified dosimeter is similar to that of the Advanced Markus chamber (AMC), manufactured by PTW‐Freiburg GmbH (Freiburg, Germany), enabling its use in typical experimental setups with commercial water phantoms or solid water phantoms. The alanine pellets and radiochromic films were calibrated under electron and proton CONV beams, traceable to the Co‐60 gamma‐ray absorbed‐dose‐to‐water, which is the reference field of the Korea Research Institute of Standards and Science (KRISS), the NMI of the Republic of Korea.^29^ The uncertainty in the doses measured by the alanine and radiochromic films was thoroughly evaluated. Under the proposed approach, the unified dosimeter enables cross‐validation of FLASH RT facilities, improving the consistency and reliability of dosimetric measurements across different facilities.

## 2 METHODS

### 2.1 Reference dose‐response curve for the alanine dosimeter

An electron spin resonance (ESR) spectrometer (JES‐FA‐100, JEOL, Japan) was employed to measure the stable radiation‐induced signals (RIS) generated in the alanine dosimeters.^30^ The absorbed dose to water, measured using the alanine dosimeters for both the electron and proton FLASH beam, was traceable to the reference beam quality at Co‐60, denoted as $Q_{0}$. The absorbed dose to water for $Q_{0}$, $D_{w,Q_{0}}^{ch}$, was initially calibrated employing a Farmer‐type ionization chamber (TW30013, S/N 8979, PTW‐Freiburg GmbH, Freiburg, Germany). This calibration was performed using a Co‐60 irradiator (AECL Eldorado 8) installed at KRISS. A water phantom was positioned with its surface aligned at a source‐to‐surface distance (SSD) of 100 cm, with the reference depth for $Q_{0}$ set at 5 g/cm^2^. The absorbed dose to water is measured by the ionization chamber for the beam quality $Q_{0}$, $D_{w,Q_{0}}^{ch}$, is given by Equation (1), where $M_{Q_{0}}^{ch}$ represents the corrected reading obtained from the ionization chamber, and $N_{D_{w,}Q_{0}}^{ch}$ denotes the absorbed dose‐to‐water calibration coefficient for the ionization chamber. The absorbed dose to water measured by the ionization chamber for beam quality Q, $D_{w,Q}^{ch}$, can be expressed by Equation (2):

(1) $$D_{w,Q_{0}}^{ch}=M_{Q_{0}}^{ch}N_{D_{w,}Q_{0}}^{ch}$$

(2) $$D_{w,Q}^{ch}=M_{Q}^{ch}k_{Q,Q_{0}}^{ch}N_{D_{w,}Q_{0}}^{ch}$$

where $M_{Q}^{ch}$ denotes the corrected chamber reading obtained from the ionization chamber, $k_{Q,Q_{0}}^{ch}$ represents the beam quality conversion factor that accounts for differences between $Q_{0}$ and Q, and $N_{D_{w,}Q}^{ch}$ indicates the absorbed dose‐to‐water calibration coefficient of the ionization chamber.

Following the IAEA TRS‐398 guidelines, a formula was derived to calculate the absorbed dose to water for alanine dosimetry.^31^ The absorbed dose to water for the reference beam quality $Q_{0}$, $D_{w,Q_{0}}^{Alan}$, and for the beam quality Q, $D_{w,Q}^{Alan}$, are determined using Equations (3)–(5):

(3) $$D_{w,Q_{0}}^{Alan}=M_{Q_{0}}^{Alan}\;N_{D_{w,}Q_{0}}^{Alan}$$

(4) $$D_{w,Q}^{Alan}=M_{Q}^{Alan}k_{Q,Q_{0}}^{Alan}N_{D_{w,}Q_{0}}^{Alan}=M_{Q}^{Alan}N_{D_{w,}Q}^{Alan}$$

(5) $$k_{Q,Q_{0}}^{Alan}=\frac{N_{D_{w},Q}^{Alan}}{N_{D_{w},Q_{0}}^{Alan}}$$

where $D_{w,Q_{0}}^{Alan}$ denotes the absorbed dose to water measured by the alanine dosimeter, $M_{Q_{0}}^{Alan}$ represents the signal obtained from the alanine dosimeter, and $N_{D_{w,}Q_{0}}^{Alan}$ indicates the absorbed dose‐to‐water calibration coefficient of the alanine dosimeter for beam quality *Q*. Likewise, $D_{w,Q}^{Alan}$ denotes the absorbed dose to water measured by the alanine dosimeter, $M_{Q}^{Alan}$ represents the signal obtained from the alanine dosimeter, and $N_{D_{w,}Q}^{Alan}$ indicates the absorbed dose‐to‐water calibration coefficient of the alanine dosimeter for beam quality *Q*. Assuming $D_{w,Q_{0}}^{ch}=D_{w,Q_{0}}^{Alan}\;$ and $D_{w,Q}^{ch}=D_{w,Q}^{Alan}\;,$ $k_{Q,Q_{0}}^{Alan}\;$ for the alanine dosimeter can be derived experimentally using Equation (5). The factor $k_{Q,Q_{0}}^{Alan}$ adjusts for the difference in the alanine response between the reference beam ($Q_{0}$) and beam quality (Q).

$M_{Q}^{Alan}$ is the measured value from the alanine dosimeter irradiated by $D_{w,Q}^{Alan}$ in beam quality Q, and it is determined as shown in Equation (6):

(6) $$M_{Q}^{Alan}=\left(\frac{A_{Q}^{Alan}}{A_{Q_{0},std}^{Alan}/D_{w,Q_{0},std}^{ch}}\right)$$

where $A_{Q}^{Alan}$ denotes the amplitude of the RIS of the alanine dosimeter irradiated by a dose of $D_{w,Q}^{ch}$ with beam quality Q, and $A_{Q_{0},std}^{Alan}$ represents the amplitude of the RIS of the alanine dosimeter irradiated by the dose of $D_{w,Q_{0},std}^{ch}$ with beam quality $Q_{0}$.^32^, ^33^, ^34^ The standard condition (denoted as ‘*std’* in Equation 6) is defined as 60 Gy delivered by the Co‐60 reference beam.

A dose–response curve was established between $D_{w,Q_{0}}^{Alan}$ and $M_{Q_{0}}^{Alan}$ for $Q_{0}$ in the dose range of 10–100 Gy (10, 30, 60, and 100 Gy). A polyoxymethylene (POM) holder was used to simulate the Farmer‐type ionization chamber, with a density of 1.41 g/cm^3^ and a thickness of 1 mm. Commercial alanine pellets (Harwell dosimeter, Goleta, CA, USA) from the same batch (no. CA600) were employed. These pellets were stored under specified humidity conditions (< 40%) for at least 8 weeks before irradiation.^35^ Additionally, the amplitude of the background spectra from 20 unirradiated alanine pellets was included in the establishment of the dose–response curve.

The measurement settings for the ESR readings were as follows: a sweep time of 60 s, time constant of 0.1 s, microwave power of 2.4 mW, modulation amplitude of 0.7 mT, and sweep width of 20 mT. The standard uncertainty and its contributing sources of $D_{w,Q}^{Alan}$ were assessed according to JCGM 100:2008.^36^

### 2.2 Electron FLASH beam

#### 2.2.1 Calibration of the alanine dosimeter with the CONV electron beam

The $k_{Q,Q_{0}}^{Alan}$ for the CONV electron beam was obtained using Equation (5). An alanine holder made of POM (mentioned in Section 2.1) was employed. The $N_{D_{w},Q}^{\mathrm{Alan}}$ was derived based on the updated formalism of AAPM WGTG51 Report 385, with dose calibration performed using a Farmer‐type ionization chamber (TW30013, S/N9304).^37^ The $N_{D_{w},Q}^{\mathrm{Alan}}$ for electron beams was determined from three electron energies (6, 12, and 18 MeV) using the Elekta Synergy Platform (Elekta AB, Stockholm, Sweden). The average value of $k_{Q,Q_{0}}^{Alan}\;$ for these energies was subsequently used as the beam quality conversion factor. Using Equation (4), the dose of the electron FLASH beam was obtained from the measured $M_{Q}^{Alan}$ and electron beam quality conversion factor.

#### 2.2.2 Unified alanine and film dosimeter for the electron FLASH beam

We developed the KRISS unified ALanine‐and‐Film dosimeter (ALF) to replicate the commercial AMC parallel plate ionization chamber. The outer dimensions of the ALF dosimeter match those of the AMC, enabling easy replacement of the AMC with the ALF dosimeter in commercial water phantom setups. As the AMC has been widely used for FLASH beam dosimetry, this interchangeability between the AMC and ALF dosimeter provides a significant advantage for dosimetry practices.

**FIGURE 1:**
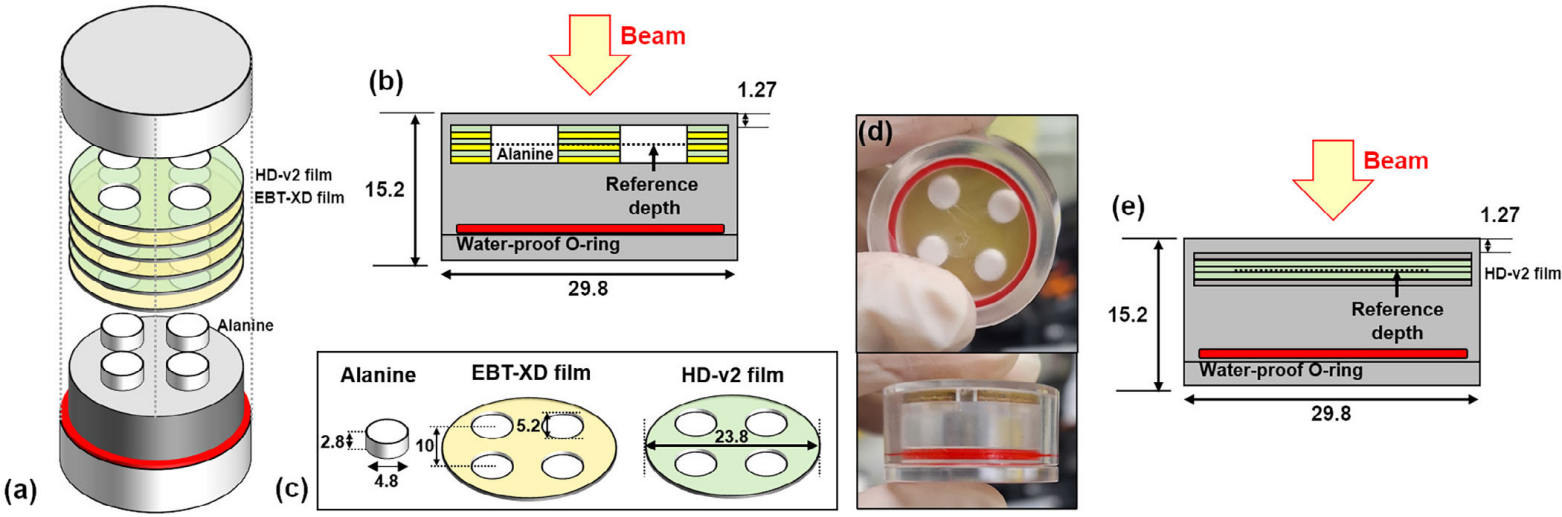
(a) Unified ALanine‐and‐Film dosimeter (denoted as ALF dosimeter) used for electron FLASH beam dosimetry. (b) Cross‐sectional view of the ALF dosimeter. (c) Dimensions of the alanine pellet, EBT‐XD film, and HD‐V2 film. (d) Photograph of the ALF dosimeters. (e) Uniformity correction dosimeter with HD‐V2 films inserted. The outer dimensions of the uniformity correction dosimeter are identical to those of the ALF dosimeter. All measurements in the figure are in millimeters (mm).

The ALF dosimeter comprises four alanine pellets and three pieces each of EBT‐XD and HD‐V2 films. The four alanine pellets are positioned at the top, bottom, left, and right of the dosimeter. The EBT‐XD and HD‐V2 films were stacked sequentially, as shown in Figure 1a,b. A laser cutting machine (Coryart, Republic of Korea) was used to cut the EBT‐XD and HD‐V2 films into circular disks, as shown in Figure 1c. The detailed dimensions of the alanine pellets and films, along with photographs of the ALF dosimeter, are also depicted in Figure 1c,d. The dose at the central region of the ALF dosimeter was assessed for both the EBT‐XD and HD‐V2 films.

As the four alanine pellets were not located at the center of the dosimeter, a uniformity correction was applied to derive the dose‐to‐alanine at the central region. For this correction, a dosimeter with the same outer dimensions as the ALF dosimeter but without alanine pellets was used. Three pieces of HD‐V2 films were inserted into this uniformity correction dosimeter, as depicted in Figure 1e. The dose values measured by the three HD‐V2 films at the four positions (top, bottom, left, and right) were averaged. The uniformity correction factor was defined as the ratio of this averaged dose to the dose at the central region. This correction factor was subsequently applied to the averaged dose across the four alanine pellets to determine the dose at the central region of the ALF dosimeter.

#### 2.2.3 Calibration of the EBT‐XD and HD‐V2 films

The EBT‐XD and HD‐V2 films were calibrated using the electron beam of the Elekta Synergy Platform against the Farmer‐type ionization chamber traceable to the absorbed dose to water of the Co‐60 reference field at KRISS. The CONV electron beam had an energy of 12 MeV, with a reference depth of 2.71 cm. The field size was 10 cm × 10 cm, and the SSD was 100 cm. 2.4‐cm‐thick solid water slabs were placed above the ALF dosimeter, while 10 cm of solid water slabs were positioned below it. The water‐equivalent depth (WED) at the measurement point for the alanine pellets was 2.71 cm. Doses corresponding to 494, 790, 1284, 2070, 3410, 5530, and 8890 monitor units (MUs) were delivered. Three unirradiated pieces of each EBT‐XD and HD‐V2 film were used to subtract background pixel values. The films were scanned 24 h after irradiation using a 10000XL flatbed scanner (Seiko Epson Corp., Japan) in transmission mode at a resolution of 300 dpi. The films were scanned at a consistent location on the scanner bed using a laser‐cut polyethylene terephthalate (PET) sheet jig designed to hold the films in the fixed positions. The region of interest (ROI) for analysis was defined as the central region of the film, with a diameter of 5 mm. The pixel values of the red channel for both EBT‐XD and HD‐V2 films were extracted using ImageJ software (ver. 1.54 g, NIH, USA) and used to derive the optical density values. A lateral response artifact for the EBT‐XD films was corrected by the method proposed by Lewis and Chan.^38^ A third‐degree polynomial function was applied to fit the relationship between net optical density and delivered dose. All film dosimetry procedures followed the protocol published by AAPM TG 235.^39^

#### 2.2.4 Electron FLASH beam irradiation

**FIGURE 2:**
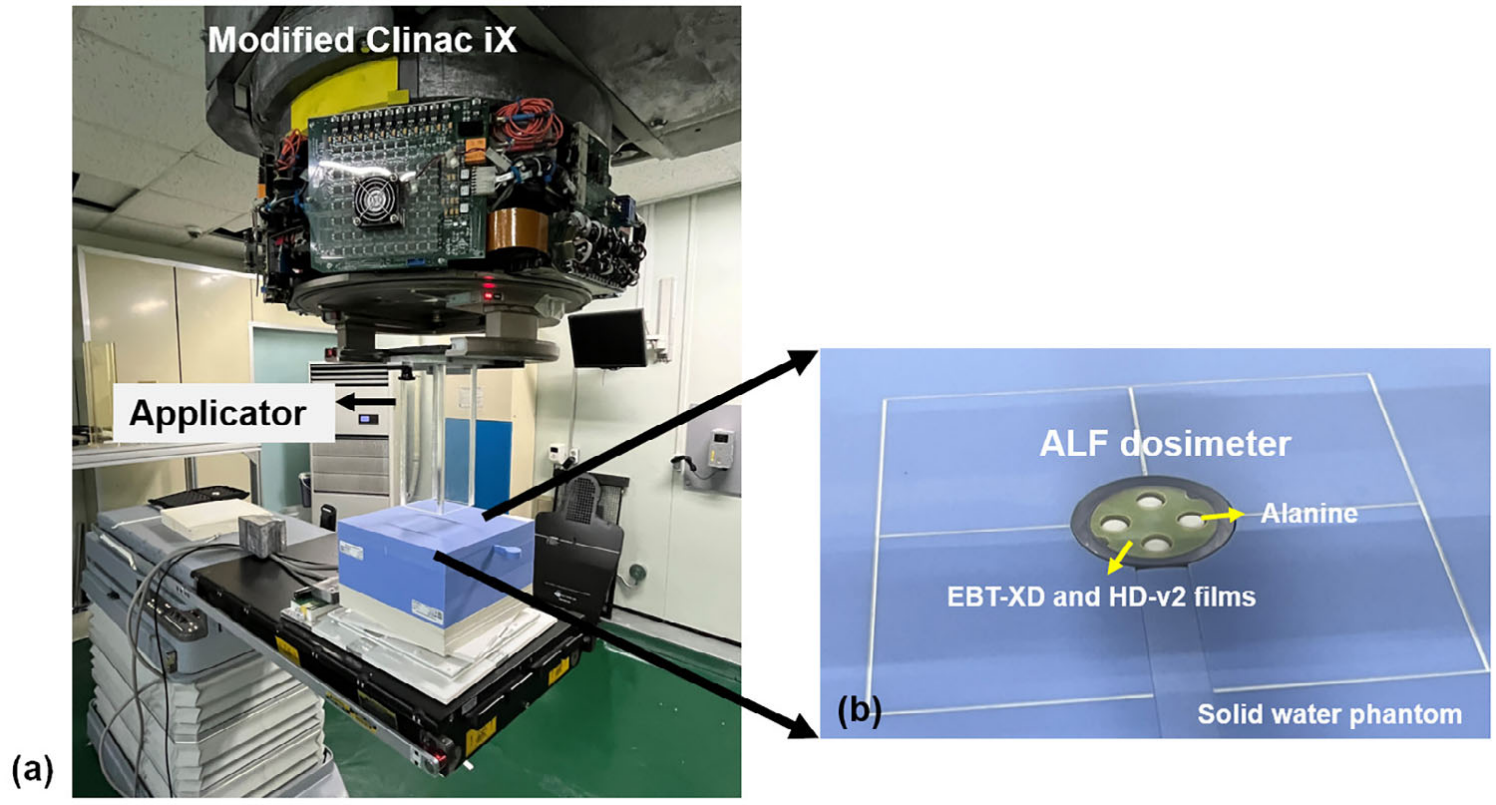
(a) Modified Varian Clinac iX for electron FLASH RT and (b) the unified Alanine‐and‐Film (ALF) dosimeter inserted in a commercial solid water phantom slab (Sun Nuclear). As the ALF dosimeter shares the same dimensions with the PTW AMC, it can easily replace the AMC for measurements. AMC, advanced Markus chamber.

Electron FLASH beam delivery was conducted using a Clinac iX (Varian Medical Systems, Palo Alto, USA) installed at the Korea Institute of Radiological & Medical Sciences (KIRAMS), Republic of Korea, as shown in Figure 2. The CONV 10 MV X‐ray beam was modified into a 9 MeV electron FLASH beam by removing the target and the flattening filter. To empty the carousel port, manual operation mode was required for the carousel board, and several functions were overridden through the service mode of the Varian treatment delivery system. The pulse width was 4 μs, with a repetition rate of 360 Hz. A 10 cm × 10 cm field size was created using an in‐house polymethylmethacrylate (PMMA) cone. The reference depth for the 9 MeV electron FLASH beam was 2.20 cm. Percent depth dose measurements were performed using EBT‐XD films. A 2‐cm‐thick solid water slab was placed above the ALF dosimeter, with 10‐cm‐thick solid water slabs positioned below it. The WED at the point of measurement was 2.31 cm, and the SSD was set to 100 cm. Two sets of irradiations were delivered, with beam pulses of 12, 22, 31, and 49.

### 2.3 Proton FLASH beam

#### 2.3.1 Calibration of the alanine dosimeter with the CONV proton beam

A cross‐calibration of the spread‐out‐Bragg‐peak (SOBP) proton beam, with a residual range of 2.5 cm, was conducted using the AMC (SN2342, PTW‐Freiburg, Freiburg, Germany) against Co‐60, with a Farmer‐type chamber (TW30013, SN9304), based on the revised version of TRS‐398.^31^ The cross‐calibration of AMC for dose measurements was performed at a depth of 17.5 g/cm^2^ in the SOBP region against the Farmer‐type chamber using the Proteus 235 proton therapy system (IBA, Belgium).^34^ The absorbed dose to water at 2 g/cm^2^ for the CONV proton single‐Bragg‐peak (SBP) beam, with beam quality Q (SBP beam), was obtained through cross‐calibration between the beam qualities *Q_cross_* (SOBP beam) and *Q* (SBP beam).

Similar to the derivation of $k_{Q,Q_{0}}^{Alan}$ for the electron beam, the corresponding factor $k_{Q,Q_{0}}^{Alan}$ for the proton SBP beam was obtained using Equations (3)–(5), assuming that $D_{w,Q}^{ch}$ corresponds to $D_{w,Q}^{Alan}$.^34^ To ensure that the dose delivered to the alanine dosimeter ($D_{w,Q}^{Alan}$) equaled $D_{w,Q}^{ch}$, an alanine holder made of PMMA was used to mimic the AMC. Using Equation (4), the dose of the proton FLASH beam was obtained from the measured $M_{Q}^{Alan}$ and proton SBP beam quality conversion factor.

#### 2.3.2 Unified alanine and film dosimeter for the proton FLASH beam

**FIGURE 3:**
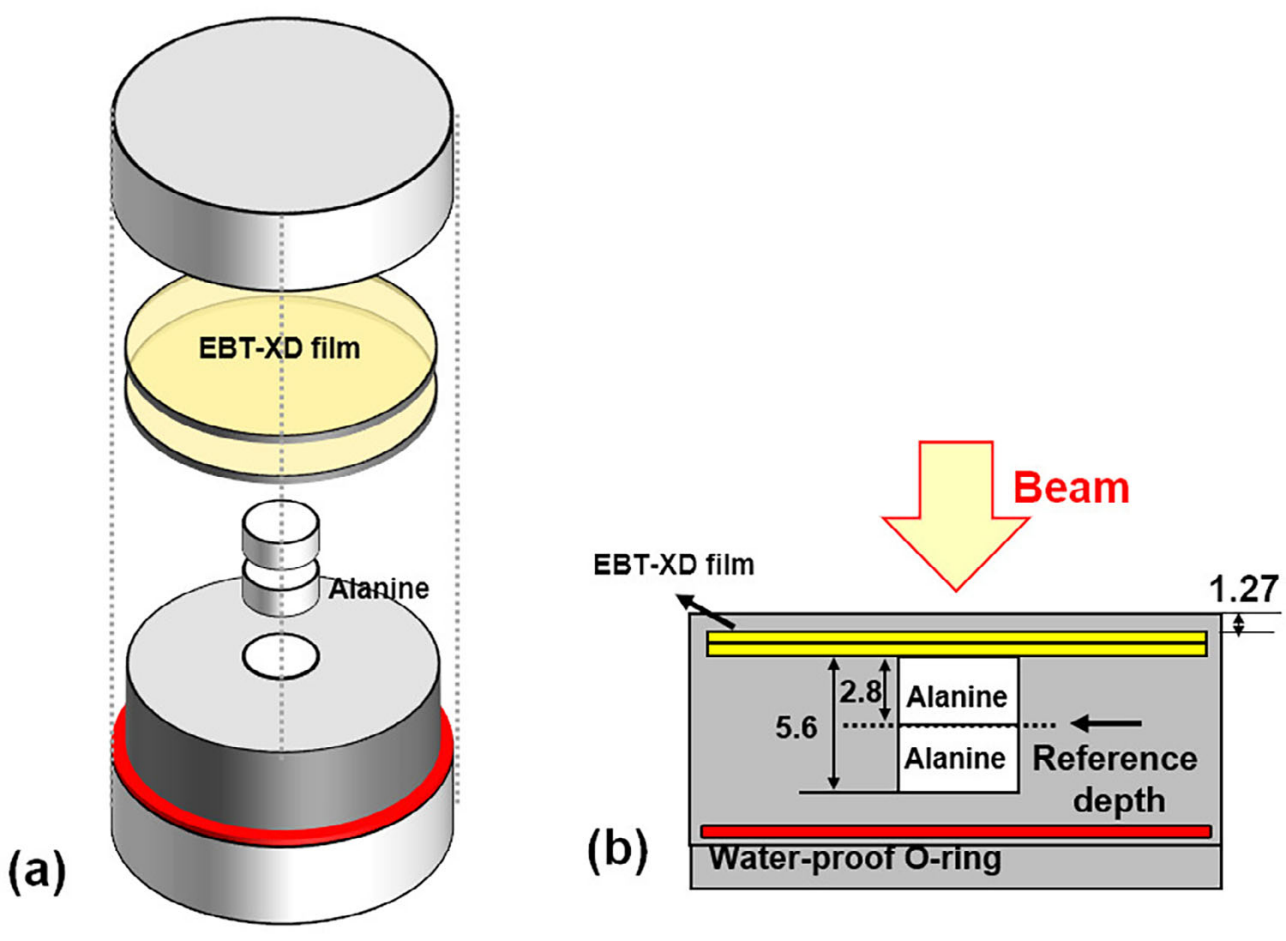
(a) Unified ALanine‐and‐Film dosimeter used for Small‐field proton FLASH beam dosimetry (denoted as the ALFS dosimeter). (b) Cross‐sectional view of the ALFS dosimeter. The unit of values in the figure is mm. The outer dimensions of the ALFS dosimeter are the same as those of the ALF dosimeter. ALF, ALanine‐and‐Film.

We designed the ALanine‐and‐Film dosimeter for Small‐field proton FLASH beam (ALFS dosimeter) to replicate the commercial AMC parallel plate ionization chamber. The outer dimensions of the ALFS dosimeter are identical to those of the ALF dosimeter and AMC. As a proton FLASH beam typically involves a small field, the ALFS dosimeter was designed with: two alanine pellets arranged in a line parallel to the beam direction, two pieces of EBT‐XD films placed above the alanine pellets, and the alanine pellets positioned at the central region of the ALFS dosimeter. The detailed dimensions of the ALFS dosimeter are shown in Figure 3. To account for the non‐uniform dose distribution in the small‐field proton FLASH beam, two‐dimensional data from the EBT‐XD films were used to derive the dose‐to‐alanine ratio. A small field correction factor was applied to compensate for variations in dose delivery to the alanine pellets. We analyzed the radial dose profiles at 0°–330° in 30° intervals (i.e., 12 radial dose profiles for each film × two pieces of film × multiple irradiations at each dose level × *N* dose levels). The dose at the center of each profile was normalized to one. *N* was 7 for the calibration process using the CONV proton beam and 4 for the FLASH beam irradiation. The radial dose profiles were averaged for both the CONV proton beam and the FLASH proton beam. The mean dose for each proton beam type was calculated by averaging the dose within a 0–2.5 mm radius, which corresponds to the radius of the alanine pellet. The small field correction factor was then calculated by taking the reciprocal of the mean dose. The experiment was conducted using an in‐house developed water phantom for proton FLASH beam dosimetry. Although the ALF and ALFS holders were designed to be waterproof and successfully protected alanine dosimeters, preliminary tests with HD‐V2 films revealed occasional water ingress due to capillary action along the holder wall. Because the active layer of HD‐V2 lacks a protective substrate, we excluded it from water‐phantom measurements in this study. A redesigned holder incorporating a water‐trapping groove is under development.

#### 2.3.3 Calibration of the EBT‐XD films

The EBT‐XD films were calibrated in the proton CONV pencil beam (Proteus 235, IBA, Belgium) against the AMC traceable to the reference field of Co‐60 at KRISS. Two sets of irradiation were conducted, with the difference in delivered MUs between the two sets being negligible. Consequently, we averaged the results from both sets for a single irradiation dose. We delivered 186.6, 298.0, 484.2, 782.3, 1265.1, 2008.5, and 3307.8 MU to the ALFS dosimeter. Four pieces of unirradiated EBT‐XD films were used to subtract the background pixel values. The films were scanned 24 h after irradiation using the 10000XL flatbed scanner in transmission mode with a resolution of 300 dpi. The ROI for dose assessment was defined as the central region of the film, with a 5 mm diameter. Pixel values from the red channel for the EBT‐XDs were extracted using ImageJ software and were used to derive the optical density. A third‐degree polynomial function was applied to model the relationship between net optical density and delivered dose.

#### 2.3.4 Proton FLASH beam irradiation

**FIGURE 4:**
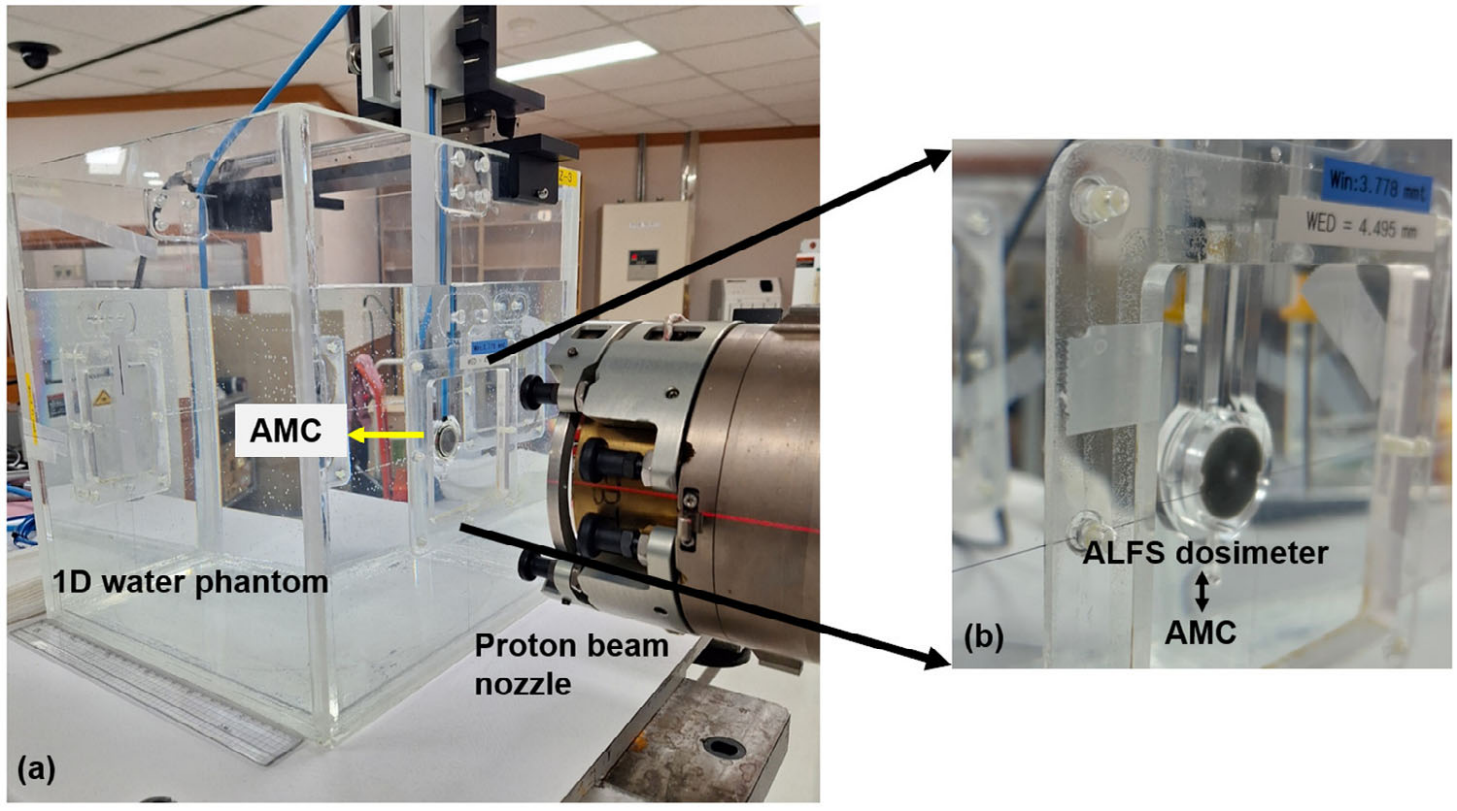
(a) Modified IBA proton therapy for proton FLASH beam. (b) The unified ALanine‐and‐Film dosimeter for Small‐field proton FLASH beam dosimetry (denoted as the ALFS dosimeter in this study) inserted into an in‐house‐developed water phantom. As the ALFS dosimeter shares the same dimensions as the AMC, it can easily replace the AMC. AMC, advanced Markus chamber.

Proton FLASH beam delivery was carried out using the Proteus 235 (IBA, Belgium) installed at the Proton Therapy Center, National Cancer Center (NCC), Republic of Korea, as depicted in Figure 4. The nominal 230 MeV single Bragg peak beam (CONV proton beam) was converted into a proton FLASH beam by removing the range modulator. A 60‐μm‐thick lead foil was used to scatter the pencil beam. The beam current was set to 200 nA, and beam delivery was controlled by an in‐house time control system using a gating signal. Two sets of irradiation were delivered to the ALFS dosimeter at a reference depth (*z*_ref_) of 2.0 g/cm^2^. The requested delivery periods were 88.7, 177.4, 266.1, and 354.8 ms. In this study, we ignored the depth dose difference between the two alanine pellets as well as the difference between the EBT‐XD films and the alanine pellets. The percent depth dose difference between the two alanine pellets was within 0.2%p, and the difference between the midpoint of the EBT‐XD films and the midpoint of the alanine pellets was within 0.1%p.

## 3 RESULTS

### 3.1 Calibration curves

The uniformity correction factors for the CONV electron and FLASH electron beams were 1.001 and 1.003, respectively. The electron FLASH beam produced an almost uniform dose distribution, with variations within 0.3% in a circular region of 2.5 cm diameter at an SSD of 100 cm and at the reference depth. However, the small‐field correction factors for the CONV proton and FLASH proton beams were 1.003 and 1.014, respectively. The dose at the central region of the FLASH proton beam was 1.4% higher than that of the average dose over a 2.5‐cm diameter.

**FIGURE 5:**
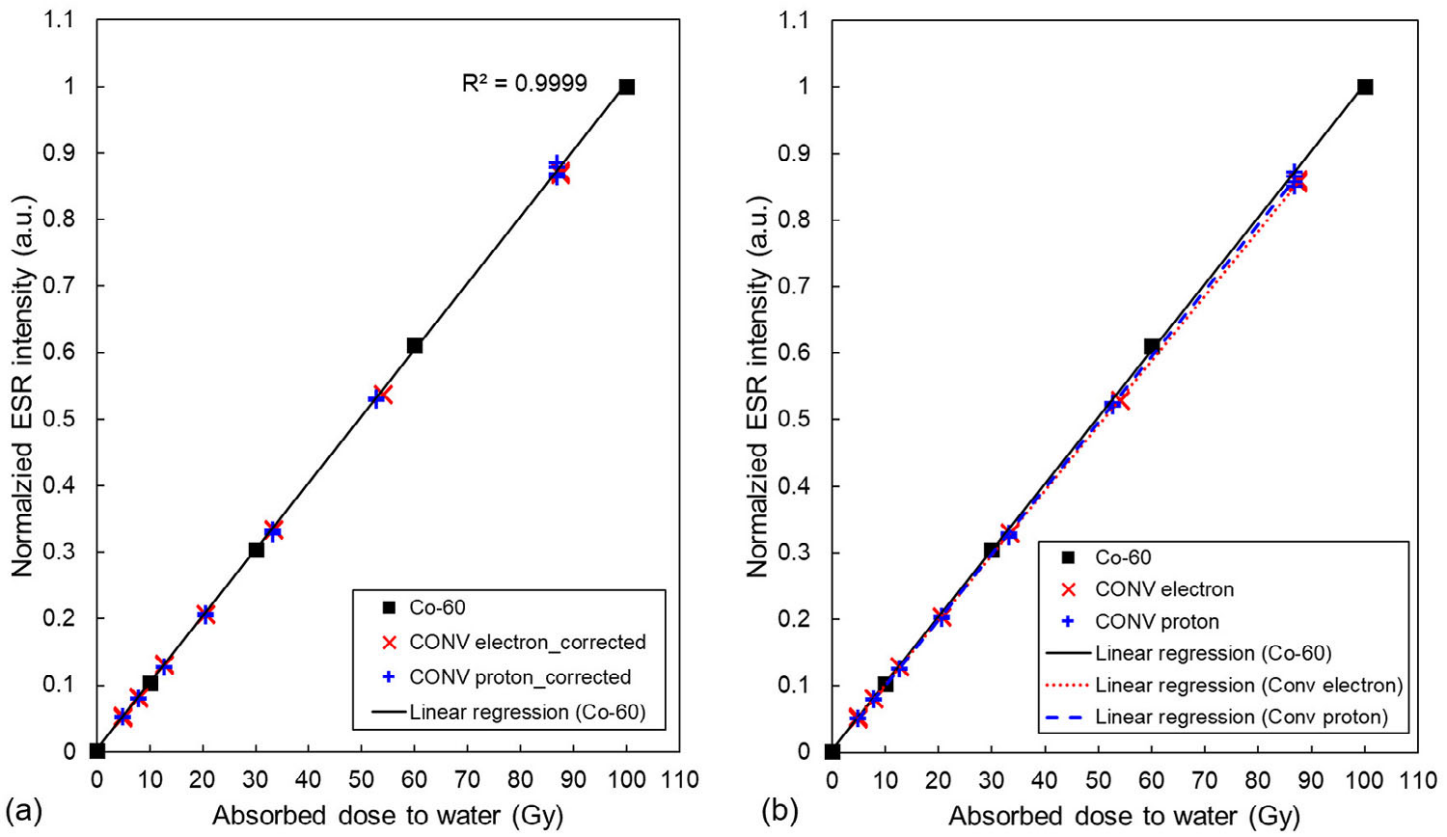
(a) Dose–response curve of alanine pellets for Co‐60 gamma‐ray absorbed dose to water. The normalized electron spin resonance (ESR) intensity, corrected by the beam quality conversion factor, $k_{Q,Q_{0}}^{Alan}$ for electron and proton beams, lies on the linear regression curve for Co‐60. (b) Dose‐response curve of alanine pellets for Co‐60 gamma‐ray, electron, and proton beams before the beam quality correction.

Figure 5 shows the dose–response curve for the reference Co‐60 gamma rays, ranging from 0 to 100 Gy. The red (x) and blue (+) markers denote the normalized ESR intensity, corrected by $k_{Q,Q_{0}}^{Alan}$. The corrected values align with the linear regression curve for the Co‐60 gamma‐ray dose–response, demonstrating that $k_{Q,Q_{0}}^{Alan}$ values for both the electron and proton beams were correctly derived. The $k_{Q,Q_{0}}^{Alan}$ was found to be 1.014 for both the electron and proton beams. The $k_{Q,Q_{0}}^{Alan}\;$ was the same level with the previous work derived at 3 cm of plateau region with the same proton beam quality.^34^

**FIGURE 6:**
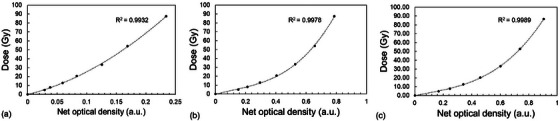
Dose–response curves for (a) HD‐V2 films for conventional (CONV) electron beams, (b) EBT‐XD films for CONV electron beams, and (c) EBT‐XD films for CONV proton beams. The pixel values of the red channel for both EBT‐XD and HD‐V2 films were used. The calibration curves were fitted using third‐degree polynomial functions.

The dose–response curves between net optical density and dose for the EBT‐XD and HD‐V2 films are presented in Figure 6. The dose values were determined using $\;D_{w,Q}^{\mathrm{ch}}$. All three fitting curves exhibited a strong correlation, with *R* ^2^ > 0.9932.

### 3.2 FLASH beam dosimetry

**FIGURE 7:**
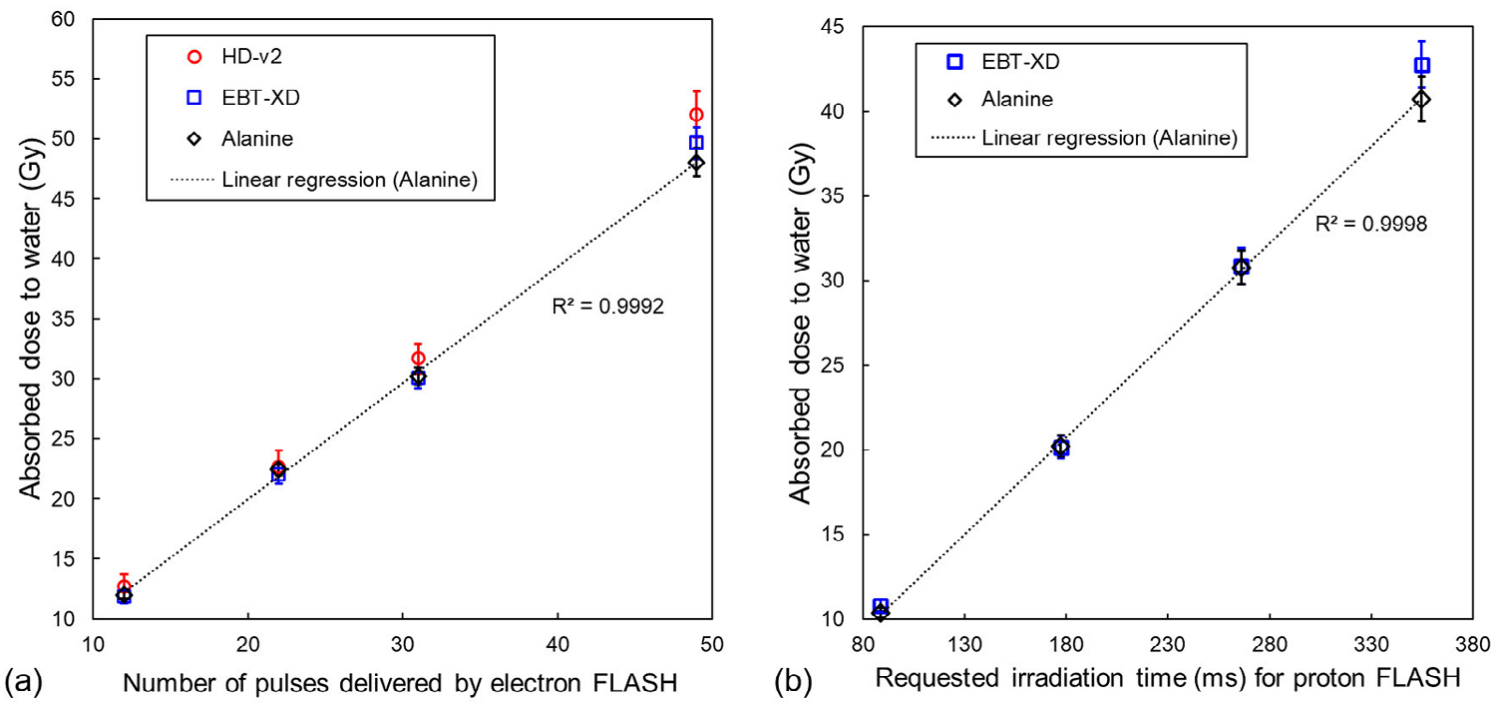
(a) Absorbed dose to water as a function of the number of pulses delivered by the electron FLASH beam and (b) Absorbed dose to water as a function of the requested irradiation time for the proton FLASH beam. The error bars indicate the combined standard uncertainty (*k* = 1).

Figure 7a illustrates the absorbed dose to water measured by the ALF dosimeters for electron FLASH beams with 12, 22, 31, and 49 pulses. The alanine dosimeters exhibited a strong linear relationship between delivered pulses and dose (*R* ^2^ > 0.9992). The absorbed dose to water of electron FLASH beams measured by alanine, HD‐V2, and EBT‐XD films is listed in Table 1. The relative dose differences between the HD‐V2 films and alanine dosimeters were 5.9%, 0.7%, 4.8%, and 8.3% for 12, 22, 31, and 49 pulses, respectively. For the EBT‐XD films, the relative dose differences were −1.2%, −2.0%, −0.5%, and 3.3%, for the same pulse counts.

**TABLE 1:** Absorbed dose to water of the electron FLASH beam measured by alanine, HD‐V2, and EBT‐XD films.

	Absorbed dose to water							
	Alanine		HD‐V2 film			EBT‐XD film		
Electron beam pulse	Dose (Gy)	Type A uncertainty (%)	Dose (Gy)	Type A uncertainty (%)	Relative difference ^a^(%)	Dose (Gy)	Type A uncertainty (%)	Relative difference ^b^(%)
12	12.0	0.4	12.7	6.5	5.9	11.8	0.8	−1.2
22	22.5	0.6	22.7	4.2	0.7	21.9	1.6	−2.0
31	30.2	0.6	31.7	2.9	4.8	30.0	0.6	−0.5
49	48.1	0.4	51.1	1.9	8.3	49.6	1.1	3.3

^a^ Relative difference between the dose measured by alanine pellets and HD‐V2 films.

^b^ Relative difference between the dose measured by alanine pellets and EBT‐XD films.

Figure 7b illustrates the absorbed dose to water measured by the ALFS dosimeters for the proton FLASH beams at irradiation times of 88.7, 177.4, 266.1, and 354.8 ms. The alanine dosimeters showed a strong linear relationship between irradiation time and dose (*R* ^2^ > 0.9998). The absorbed dose to water of proton FLASH beams measured by alanine and EBT‐XD films is listed in Table 2. The relative dose differences between the EBT‐XD films and alanine dosimeters were 4.3%, −0.4%, 0.3%, and 5.1% for irradiation times of 88.7, 177.4, 266.1, and 354.8 ms, respectively.

**TABLE 2:** Absorbed dose to water of the proton FLASH beam measured by alanine and EBT‐XD films.

	Absorbed dose to water				
	Alanine		EBT‐XD film		
Proton beam irradiation time (ms)	Dose (Gy)	Type A uncertainty (%)	Dose (Gy)	Type A uncertainty (%)	Relative difference^a^ (%)
88.7	10.4	0.2	10.8	0.5	4.3
177.4	20.2	0.8	20.1	0.9	−0.4
266.1	30.8	0.4	30.9	1.9	0.3
354.8	40.7	0.4	42.8	1.4	5.1

^a^ Relative difference between the dose measured by the alanine pellets and the EBT‐XD films.

The dose per pulse for the electron FLASH beams and the mean dose rate for the proton FLASH beams are listed in Table 3. The instantaneous dose rate for the electron FLASH beams measured by the alanine dosimeters was calculated as 2.48 × 10^5^ Gy/s, and the mean dose rate was 357 Gy/s.

**TABLE 3:** Dose per pulse of the electron FLASH beam and mean dose rate of the proton FLASH beam measured by ALanine‐and‐Film (ALF) and ALanine‐and‐Film for Small field (ALFS) dosimeters.

	Dose per pulse of electron FLASH beam (Gy/pulse)				Mean dose rate of proton FLASH beam (Gy/s)	
Electron beam pulse	Alanine	HD‐V2	EBT‐XD	Proton beam irradiation time (ms)	Alanine	EBT‐XD
12	1.00	1.06	0.99	88.7	117.1	122.2
22	1.02	1.03	1.00	177.4	114.1	113.7
31	0.98	1.02	0.97	266.1	115.6	116.0
49	0.97	1.05	1.00	354.8	114.7	120.6
Average	0.99 ±0.02	1.04 ±0.01	0.99 ±0.01	Average	115.4 ±1.1	118.1 ±3.4

### 3.3 Uncertainty budgets

Table 4 lists the sources of uncertainty and their respective uncertainties for the absorbed dose to water of electron FLASH beams at 22 pulses. The combined relative standard uncertainty (*k* = 1) was 2.4% for alanine dosimeters, 3.5% for EBT‐XD films, and 6.0% for HD‐V2 films. Table 5 lists the uncertainty sources and their respective uncertainties for the absorbed dose to water of proton FLASH beams at 177.4 ms irradiation. The combined relative standard uncertainty (*k* = 1) was 3.2% for both alanine dosimeters and EBT‐XD films.

**TABLE 4:** Uncertainty budgets for the absorbed dose to water of the electron FLASH beam at 22 pulses.

		Relative standard uncertainty (%)			
		Film		Alanine	
Physical quantity or procedure	Description for uncertainty sources	Type A	Type B	Type A	Type B
**Step 1: Reference dosimetry**					
$N_{D_{w},Q_{0}}$	$N_{D_{w}}$ calibration at the beam quality $Q_{0}$		0.5		0.5
$k_{Q,Q_{0}}^{ch}$ or $k_{Q,Q_{0}}^{Alan}$	Beam quality correction for chamber		0.7		
	Beam quality correction for alanine				0.8
$M_{Q}^{ch}$ or $M_{Q}^{Alan}$	Dosimeter reading from chamber or alanine	0.1		0.3	
	Corrections for chamber or alanine		0.3		0.6
$D_{w,Q}^{ch}$ or $D_{w,Q}^{Alan}$	Combined uncertainty for Step 1	0.9		1.2	
**Step 2: Dose response calibration**					
Calibration of film or alanine	Curve fitting for film^a^		3.7 (HD‐V2) 2.1 (EBT‐XD)		
	Flatbed scanner uniformity		0.5		
	ESR spectrometer drift				0.5
	Combined uncertainty in Steps 1 and 2	2.3		1.3	
**Step 3: FLASH beam dosimetry**					
FLASH beam	Flash beam stability		2.0		2.0
	Dosimeter reading of film or alanine	4.2 (HD‐V2) 1.6 (EBT‐XD)		0.6	
	Combined uncertainty in Steps 1, 2, and 3 (*k* = 1)	**6.0** (HD‐V2) **3.5** (EBT‐XD)		**2.4**	

^a^ The uncertainty for the curve fitting was evaluated based on JCGM 100:2008.^36^

**TABLE 5:** Uncertainty budgets for the absorbed dose to water of the proton FLASH beam at 177.4 ms irradiation.

		Relative standard uncertainty (%)			
		Film		Alanine	
Physical quantity or procedure	Description for uncertainty sources	Type A	Type B	Type A	Type B
**Step 1: Reference dosimetry**					
$N_{D_{w},Q_{0}}$	$N_{D_{w}}$ calibration at the beam quality $Q_{0}$		0.5		0.5
$k_{Q,Q_{0}}^{ch}$ or $k_{Q,Q_{0}}^{Alan}$	Beam quality correction for chamber, $k_{Q_{cross},Q_{0}}^{ch}$		1.6		
	Beam quality correction for chamber, $k_{Q,Q_{cross}}^{ch}$		0.6		
	Beam quality correction for alanine				2.2
$M_{Q}^{ch}$ or $M_{Q}^{Alan}$	Dosimeter reading from chamber or alanine	0.6		0.3	
	Corrections for chamber or alanine		0.3		0.6
$D_{w,Q}^{ch}$ or $D_{w,Q}^{Alan}$	Combined uncertainty for Step 1	1.9		2.4	
**Step 2: Dose response calibration**					
Calibration of film or alanine	Curve fitting for filma		1.3		
	Flatbed scanner uniformity		0.5		
	ESR spectrometer drift				0.5
	Combined uncertainty in Steps 1 and 2	2.3		2.4	
**Step 3: FLASH beam dosimetry**					
FLASH beam	Flash beam stability		2.0		2.0
	Dosimeter reading of film or alanine	0.9		0.8	
	Combined uncertainty in Steps 1, 2, and 3 (*k* = 1)	**3.2**		**3.2**	

^a^ The uncertainty for the curve fitting was evaluated based on JCGM 100:2008.^36^

## 4 DISCUSSION

In summary, two types of dosimeters—ALF for electrons, ALFS for protons—were developed and used to measure doses in FLASH RT facilities at KIRAMS and NCC in the Republic of Korea. For electron FLASH irradiation, the alanine dosimeter measured doses with a combined relative standard uncertainty of 2.4% (*k* = 1), while the EBT‐XD films had a combined relative standard uncertainty of 3.5% (*k* = 1). The EBT‐XD films demonstrated good agreement with the alanine dosimeter, showing a relative difference within 2.0%, suggesting their suitability for routine dose measurements in the 10–30‐Gy range, considering their standard uncertainty of 3.5% (*k* = 1). However, the HD‐V2 films exhibited a relatively large difference (up to 5.9%) and combined relative standard deviation (6.0%, *k *= 1) compared to the alanine dosimeter in the 10–30‐Gy range, as their dynamic range extends to much higher doses than those examined in this study. The alanine dosimeter and EBT‐XD recorded the dose under proton FLASH irradiation with a combined relative standard uncertainty of 3.2% (*k* = 1). The EBT‐XD films demonstrated a 4.3% relative difference with the alanine dosimeter in the 10–30‐Gy range.

Since EBT‐XD and HD‐V2 films utilize almost identical active chemical components,^39^, ^40^ their structural differences significantly impact dosimetric performance. The EBT‐XD has a 25‐µm‐thick active layer, whereas HD‐V2 has a thinner 12‐µm active layer, resulting in lower sensitivity.^40^, ^41^ As a result, the EBT‐XD performs better in the dose range of this study (10–50 Gy), while HD‐V2 is designed for higher dose ranges (10–1000 Gy) and shows reduced performance at lower doses.^40^, ^41^ Moreover, the EBT‐XD's active layer is encapsulated between two 125‐µm matte polyester substrates, providing better protection from environmental factors and mechanical handling.^41^ In contrast, the active layer of HD‐V2 is exposed to air, coated only on a single 97‐µm polyester substrate.^40^ This makes HD‐V2 more susceptible to damage or signal degradation from cutting tools and environmental exposure, potentially increasing measurement uncertainty. These differences in film structure and intended dose range explain the higher type A uncertainty and reduced precision observed with HD‐V2 in this study.

The uncertainty in beam stability was a major contributor to the combined relative standard uncertainty. The beam stability of 2% (Type B) reported in Tables 4 and 5 was an estimated value based on each facility's experience. Since the beam stability uncertainty significantly contributed to the combined standard uncertainty of the FLASH dosimetry, it should be precisely assessed. We plan to perform repeated measurements using ALF and ALFS dosimeters in future work. This can also be minimized by implementing rigorous beam‐monitoring devices, such as beam current transformers.^13^, ^42^, ^43^, ^44^

The standard uncertainty of the beam quality conversion factor for the alanine dosimeters, $k_{Q,Q_{0}}^{Alan}$, for the proton beam is higher than that for the electron beam. For the proton beam, only a few studies from one group have reported the beam quality conversion factor,^45^, ^46^ compared to those for the electron beam.^47^, ^48^, ^49^, ^50^, ^51^ For the electron beam quality conversion factor, primary standard laboratories have recently proposed a single scaling factor with low uncertainty, based on previous studies.^51^

Compared to the uncertainties associated with alanine dosimeters, the curve fitting procedure for film calibration contributed more substantially to the combined relative standard uncertainty. The fitted curves exhibited strong correlation (*R* ^2^ > 0.9932), indicating good agreement. Nonetheless, we acknowledge that alternative fitting methods may further reduce the curve fitting uncertainty, and researchers may explore such options depending on their experimental objectives. The difference in uncertainty between the proton and electron dose to EBT‐XD film responses can be attributed to the impact of film preparation. In ALF, films were laser‐cut to create circular spaces for alanine pellets, potentially introducing local distortions near the cut edges. The ROI in ALF, being closer to the laser‐cut area, may have been affected by these distortions, leading to a higher measurement standard deviation (0.4%–4.0% in relative standard deviation). In contrast, ALFS used circular films without cutting for alanine pellets, resulting in fewer potential imperfections. The ROI in ALFS was less likely to be influenced by cutting‐related artifacts, leading to a more stable dose response and lower measurement standard deviations (0.3%–1.9% in relative standard deviation). This difference accounts for the lower uncertainty and better dose response curve fitting observed in ALFS. Therefore, the laser cutting process in ALF likely contributed to the increased uncertainty in the curve fitting as well as type A uncertainty in Step 3 of Tables 4 and 5.

The ALF dosimeter was designed with combined materials from films, alanine pellets, and surrounding PMMA. PTB investigated the effects of six types of alanine surrounding plastic materials, which physical density and electron density ranged from 1.04–1.819 and 1.012 to 1.692, respectively.^52^ Also, the bulk density of the alanine dosimeter and PMMA is the same level, with values of 1.16  and 1.17 g/cm^3^, respectively. The difference in measurement results when one alanine pellet and five alanine pellets are simultaneously irradiated inside the alanine dosimeter during beam irradiation was within 0.3% (this result is not included in the manuscript). For the ALFS dosimeter for the proton beam, the two pellets were serially placed in the depth direction. The previous studies showed no significant effect, Monte Carlo calculations, even for the depth dose curve with serial stacks of alanine pellets.^53^

We developed a robust alanine dosimetry protocol for the FLASH RT. Until dosimetry guidelines for the FLASH RT community are provided by a working group such as AAPM TG‐359, the dosimetry protocol proposed in this study can serve as one of the promising protocols for FLASH RT facilities worldwide, including those in the Republic of Korea. The users in the FLASH RT facilities read the calibrated film dose by themselves, while sending the alanine pellets to the KRISS. The KRISS will read the alanine dose and provide feedback to the facilities 1 week after the irradiation. This would help obtain a dosimetric consensus, and we expect to conduct a cross‐validation study based on this work.

For small field dosimetry using alanine, the two‐dimensional dose distribution measured by radiochromic film has been utilized to derive the small field correction factor (or the correction factor for volume averaging effects) for a 6 MV flattening filter‐free photon beam.^54^ The ALFS dosimeter offers the advantage of enabling simultaneous measurement of both the small field correction factor and the absorbed dose. This makes the ALFS particularly useful for small field dosimetry. Although the ALFS design could be adapted for electron beam dosimetry, its serial arrangement of alanine pellets is not suitable for the reference dosimetry in electron beams due to the dose gradient. However, its configuration is well‐suited for depth‐dose measurements. Future work will explore its application in characterizing electron beam depth‐dose distributions. The ALF and ALFS dosimeters are similar to the AMC and can be easily used with commercial water phantoms or solid water phantoms for quality assurance (QA). They can also provide the reference dose when users apply the AMC dosimetry protocol in their FLASH RT facilities. This study does not involve FLASH beam dosimetry using the AMC itself; it was only used for cross‐calibration for the CONV proton beam. The performance of the AMC for FLASH beam irradiation has already been evaluated by several other research groups.

The alanine and film dosimeters, due to their small sizes, provide a significant advantage for in vivo dosimetry. However, the ALF and ALFS dosimeters are housed in PMMA containers with fixed outer dimensions (the same as the AMC), which makes them challenging to use in vivo in their current form. A new design for in vivo dosimetry is needed and is currently under investigation. The revised in vivo ALF dosimeter will feature thinner and smaller outer dimensions, allowing for simultaneous irradiation, similar to the ALF dosimeter used in this study.

## 5 CONCLUSIONS

The unified alanine and radiochromic film dosimeters (referred to as ALF for electron beams and ALFS for proton beams) for FLASH RT dosimetry were designed and manufactured. The alanine provided the absorbed dose‐to‐water with a combined relative standard uncertainty of 2.4% (*k* = 1) for the 0.99 Gy/pulse electron FLASH beam and 3.2% (*k* = 1) for the 115.4 Gy/s proton FLASH beam. The EBT‐XD films displayed good agreement (within 2.0% and 4.3% relative differences for electron and proton beams, respectively) with the alanine dosimeter in the 10–30‐Gy range but exhibited an equal to or higher combined relative standard uncertainty (3.5% and 3.2% for electron and proton beams, respectively) compared to the alanine dosimeter. The ALF and ALFS dosimeters have the same outer dimensions as the AMC and are compatible with commercial water phantoms or solid water phantoms for QA. The dosimetry protocol proposed in this study offers a promising approach for FLASH RT facilities worldwide, including those in the Republic of Korea, until dosimetry guidelines for the FLASH RT community are established by a working group such as AAPM TG‐359.

## CONFLICT OF INTEREST STATEMENT

The author has no conflicts of interest to disclose.

## References

[1] Favaudon V , Caplier L , Monceau V , et al. Ultrahigh dose‐rate FLASH irradiation increases the differential response between normal and tumor tissue in mice. Sci Transl Med. 2014;6(245):245ra93. 10.1126/scitranslmed.3008973 25031268

[2] Bourhis J , Sozzi WJ , Jorge PG , et al. Treatment of a first patient with FLASH‐radiotherapy. Radiother Oncol. 2019;139:18‐22. 31303340 10.1016/j.radonc.2019.06.019

[3] Bourhis J , Montay‐Gruel P , Gonçalves Jorge P , et al. Clinical translation of FLASH radiotherapy: why and how?. Radiother Oncol. 2019;139:11‐17. 31253466 10.1016/j.radonc.2019.04.008

[4] Wilson JD , Hammond EM , Higgins GS , et al. Ultra‐high dose rate (FLASH) radiotherapy: silver bullet or fool's gold?. Front Oncol. 2020;9:1563. 32010633 10.3389/fonc.2019.01563 PMC6979639

[5] Gaide O , Herrera F , Jeanneret Sozzi W , et al. Comparison of ultra‐high versus conventional dose rate radiotherapy in a patient with cutaneous lymphoma. Radiother Oncol. 2022;174:87‐91. 34998899 10.1016/j.radonc.2021.12.045

[6] Daugherty EC , Mascia A , Zhang Y , et al. FLASH radiotherapy for the treatment of symptomatic bone metastases (FAST‐01): protocol for the first prospective feasibility Study. JMIR Res Protoc. 2023;12:e41812. 36206189 10.2196/41812 PMC9893728

[7] Farr JB , Parodi K , Carlson DJ . FLASH: current status and the transition to clinical use. Med Phys. 2022;49(3):1972‐1973. 35262219 10.1002/mp.15401

[8] Vozenin MC , Bourhis J , Durante M . Towards clinical translation of FLASH radiotherapy. Nat Rev Clin Oncol. 2022;19(12):791‐803. 36303024 10.1038/s41571-022-00697-z

[9] Schulte R , Johnstone C , Boucher S , et al. Transformative technology for flash radiation therapy. Appl Sci (Basel). 2023;13(8):5021. 38240007 10.3390/app13085021 PMC10795821

[10] Siddique S , Ruda HE , Chow JCL . FLASH radiotherapy and the use of radiation dosimeters. Cancers (Basel). 2023;15(15):3883. 37568699 10.3390/cancers15153883 PMC10417829

[11] Mascia AE , Daugherty EC , Zhang Y , et al. Proton FLASH radiotherapy for the treatment of symptomatic bone metastases: the FAST‐01 nonrandomized trial. JAMA Oncol. 2023;9(1):62‐69. 36273324 10.1001/jamaoncol.2022.5843 PMC9589460

[12] Montay‐Gruel P , Petersson K , Jaccard M , et al. Irradiation in a flash: unique sparing of memory in mice after whole brain irradiation with dose rates above 100 Gy/s. Radiother Oncol. 2017;124(3):365‐369. 28545957 10.1016/j.radonc.2017.05.003

[13] Subiel A , Bourgouin A , Kranzer R , et al. Metrology for advanced radiotherapy using particle beams with ultra‐high dose rates. Phys Med Biol. 2024;69(14). doi:10.1088/1361-6560/ad539d 38830362

[14] Petersson K , Jaccard M , Germond JF , et al. High dose‐per‐pulse electron beam dosimetry—a model to correct for the ion recombination in the advanced Markus ionization chamber. Med Phys. 2017;44(3):1157‐1167. 28094853 10.1002/mp.12111

[15] Vozenin MC , De Fornel P , Petersson K , et al. The advantage of FLASH radiotherapy confirmed in Mini‐pig and Cat‐cancer patients. Clin Cancer Res. 2019;25(1):35‐42. 29875213 10.1158/1078-0432.CCR-17-3375

[16] Jaccard M , Petersson K , Buchillier T , et al. High dose‐per‐pulse electron beam dosimetry: usability and dose‐rate independence of EBT3 Gafchromic films. Med Phys. 2017;44(2):725‐735. 28019660 10.1002/mp.12066

[17] Szpala S , Huang V , Zhao Y , et al. Dosimetry with a clinical linac adapted to FLASH electron beams. J Appl Clin Med Phys. 2021;22(6):50‐59. 34028969 10.1002/acm2.13270 PMC8200504

[18] Guan F , Wang X , Yang M , et al. Dosimetric response of Gafchromic^™^ EBT‐XD film to therapeutic protons. Precis Radiat Oncol. 2023;7(1):15‐26. 37868341 10.1002/pro6.1187 PMC10586355

[19] Korysko P , Bateman J , Robertson C , et al. Methods for VHEE/FLASH radiotherapy studies and high dose rate dosimetry at the clear user facility. JACoW LINAC. 2022;2022:758‐761.doi:10.18429/JACoW-LINAC2022-THPOPA06

[20] Bin J , Obst‐Huebl L , Mao JH , et al. A new platform for ultra‐high dose rate radiobiological research using the BELLA PW laser proton beamline. Sci Rep. 2022;12(1):1484. 35087083 10.1038/s41598-022-05181-3 PMC8795353

[21] Dal Bello R , von der Grün J , Fabiano S , et al. Enabling ultra‐high dose rate electron beams at a clinical linear accelerator for isocentric treatments. Radiother Oncol. 2023;187:109822. 37516362 10.1016/j.radonc.2023.109822

[22] Gu R , Wang J , Wang P , et al. Alanine/electron spin resonance dosimetry for FLASH radiotherapy. Radiat Phys Chem. 2024;225:112113.

[23] Karsch L , Beyreuther E , Burris‐Mog T , et al. Dose rate dependence for different dosimeters and detectors: TLD, OSL, EBT films, and diamond detectors. Med Phys. 2012;39(5):2447‐2455. 22559615 10.1118/1.3700400

[24] Desrosiers MF , Puhl JM , Cooper SL . An absorbed‐dose/dose‐rate dependence for the alanine‐EPR dosimetry system and its implications in high‐dose ionizing radiation metrology. J Res Natl Inst Stand Technol. 2008;113(2):79‐95. 27096113 10.6028/jres.113.007 PMC4654067

[25] León‐Marroquín EY , Mulrow D , Darafsheh A , Khan R . Response characterization of EBT‐XD radiochromic films in megavoltage photon and electron beams. Med Phys. 2019;46(9):4246‐4256. doi:10.1002/mp.13708 31297824

[26] Jorge PG , Jaccard M , Petersson K , et al. Dosimetric and preparation procedures for irradiating biological models with pulsed electron beam at ultra‐high dose‐rate. Radiother Oncol. 2019;139:34‐39. 31174897 10.1016/j.radonc.2019.05.004

[27] Gondré M , Jorge PG , Vozenin MC , et al. Optimization of alanine measurements for fast and accurate dosimetry in FLASH radiation therapy. Radiat Res. 2020;194(6):573‐579. 33348370 10.1667/RR15568.1

[28] Jorge PG , Melemenidis S , Grilj V , et al. Design and validation of a dosimetric comparison scheme tailored for ultra‐high dose‐rate electron beams to support multicenter FLASH preclinical studies. Radiother Oncol. 2022;175:203‐209. doi:10.1016/j.radonc.2022.08.023 36030934

[29] Kessler C , Burns D , Kim IJ , et al. Key comparison BIPM. RI (I)‐K4 of the absorbed dose to water standards of the KRISS, Republic of Korea and the BIPM in 60Co gamma radiation. Metrologia. 2022;59(1A):06023.

[30] Bradshaw Jr WW , Cadena DG , Crawford GW , Spetzler HA . The use of alanine as a solid dosimeter. Radiat Res. 1962;17:11‐21. 13872350

[31] INTERNATIONAL ATOMIC ENERGY AGENCY , Absorbed Dose Determination in External Beam Radiotherapy, Technical Reports Series No. 398 (Rev. 1), IAEA, 2024.

[32] Anton M , Lelie S . Alanine dosimetry—uncertainty components. PTB Bericht PTB‐Dos‐55. Physikalisch‐Technische Bundesanstalt Braunschweig und Berlin; 2010.

[33] Anton M . Uncertainties in alanine/ESR dosimetry at the Physikalisch‐Technische Bundesanstalt. Phys Med Biol. 2006;51(21):5419‐5440. 17047261 10.1088/0031-9155/51/21/003

[34] Kim CE , Park JI , Jung S , et al. Determination of beam quality correction factors for alanine dosimetry in clinical proton beams. Phys Med. 2025;134:104992. 40349668 10.1016/j.ejmp.2025.104992

[35] Arber J , Sharpe P . Fading characteristics of irradiated alanine pellets: the importance of pre‐irradiation conditioning. Appl Radiat Isot. 1993;44(1‐2):19‐22.

[36] JCGM 100:2008 . Evaluation of measurement data‐Guide to expression of uncertainty in measurement. 2008.

[37] Muir B , Davis S , Dhanesar S , et al. AAPM WGTG51 Report 385: addendum to the AAPM's TG‐51 protocol for clinical reference dosimetry of high‐energy electron beams. Med Phys. 2024;51:5840‐5857. 38980220 10.1002/mp.17277

[38] Lewis DF , Chan MF . Technical Note: on GAFChromic EBT‐XD film and the lateral response artifact. Med Phys. 2016;43(2):643‐649. 26843228 10.1118/1.4939226 PMC4715006

[39] Niroomand‐Rad A , Chiu‐Tsao ST , Grams MP , et al. Report of AAPM Task Group 235 radiochromic film dosimetry: an update to TG‐55. Med Phys. 2020;47:5986‐6025. 32990328 10.1002/mp.14497

[40] Ashland ISP Inc . GafchromicTM dosimetry media, type HD‐V2 2025. Accessed June 5, 2025 http://www.gafchromic.com/documents/gafchromic‐hdv2.pdf

[41] Ashland ISP Inc . GafchromicTM dosimetry media, type EBT‐XD 2025. Accessed June 5, 2025 http://www.gafchromic.com/documents/EBTXD_Specifications_Final.pdf

[42] Oesterle R , Gonçalves Jorge P , Grilj V , et al. Implementation and validation of a beam‐current transformer on a medical pulsed electron beam LINAC for FLASH‐RT beam monitoring. J Appl Clin Med Phys. 2021;22(11):165‐171. 10.1002/acm2.13433 PMC8598141 34609051

[43] Gonçalves Jorge P , Grilj V , Bourhis J , et al. Technical note: validation of an ultrahigh dose rate pulsed electron beam monitoring system using a current transformer for FLASH preclinical studies. Med Phys. 2022;49(3):1831‐1838. 35066878 10.1002/mp.15474 PMC9303205

[44] Liu K , Palmiero A , Chopra N , et al. Dual beam‐current transformer design for monitoring and reporting of electron ultra‐high dose rate (FLASH) beam parameters. J Appl Clin Med Phys. 2023;24(2):e13891. 36601691 10.1002/acm2.13891 PMC9924113

[45] Carlino A , Gouldstone C , Kragl G , et al. End‐to‐end tests using alanine dosimetry in scanned proton beams. Phys Med Biol. 2018;63(5):055001. 29384730 10.1088/1361-6560/aaac23

[46] Palmans H , Antonio C , Gouldstone C , Sharpe P . Cross calibration of alanine for scanned proton beams. NPL Report No. IR 48. National Physical Laboratory; 2018. https://eprintspublications.npl.co.uk/8118/

[47] Anton M , Kapsch R , Krauss A , et al. Difference in the relative response of the alanine dosimeter to megavoltage x‐ray and electron beams. Phys Med Biol. 2013;58(10):3259. 23611943 10.1088/0031-9155/58/10/3259

[48] Cronholm RO , Andersen CE , Behrens CF , Helt‐Hansen J . Volume dose ratios relevant for alanine dosimetry in small, 6 MV photon beams. Radiat Meas. 2012;47(10):1014‐1017.

[49] Hjørringgaard JG , Ankjærgaard C , Miller A , Andersen CE . Kilovoltage X‐ray beam quality effect on the relative response of alanine pellet dosemeters. Radiat Prot Dosi. 2023;199(14):1605‐1610. 10.1093/rpd/ncad008 37721066

[50] Nasreddine A , Kuntz F , El Bitar Z . Absorbed dose to water determination for kilo‐voltage X‐rays using alanine/EPR dosimetry systems. Radiat Phys Chem. 2021;180:108938.

[51] McEwen M , Miller A , Pazos I , Sharpe P . Determination of a consensus scaling factor to convert a Co‐60‐based alanine dose reading to yield the dose delivered in a high energy electron beam. Radiat Phys Chem. 2020;171:108673.

[52] Anton M , Kapsch RP , Hackel T . Is there an influence of the surrounding material on the response of the alanine dosimetry system?. Phys Med Biol. 2009;54(7):2029‐2035. 19287075 10.1088/0031-9155/54/7/011

[53] Palmans H . Effect of alanine energy response and phantom material on depth dose measurements in ocular proton beams. Technol Cancer Res Treat. 2003;2(6):579‐586. 14640769 10.1177/153303460300200610

[54] Höfel S , Liebig P , Fix MK , et al. Adapting a practical EPR dosimetry protocol to measure output factors in small fields with alanine. J Appl Clin Med Phys. 2023;24:e14191. 37922380 10.1002/acm2.14191 PMC10691647

